# TNFα affects CREB-mediated neuroprotective signaling pathways of synaptic plasticity in neurons as revealed by proteomics and phospho-proteomics

**DOI:** 10.18632/oncotarget.19428

**Published:** 2017-07-21

**Authors:** Pia Jensen, Christa L. Myhre, Pernille S. Lassen, Athanasios Metaxas, Asif M. Khan, Kate L. Lambertsen, Alicia A. Babcock, Bente Finsen, Martin R. Larsen, Stefan J. Kempf

**Affiliations:** ^1^ Department of Biochemistry and Molecular Biology, University of Southern Denmark, Odense, Denmark; ^2^ Neurobiology Research, Institute of Molecular Medicine, University of Southern Denmark, Odense, Denmark; ^3^ Department of Neurology, Odense University Hospital, Odense, Denmark; ^4^ BRIDGE, Brain Research–Inter-Disciplinary Guided Excellence, Department of Clinical Research, University of Southern Denmark, Odense, Denmark; ^5^ Current address: Department of Biochemistry and Molecular Biology, University of Southern Denmark, Odense, Denmark

**Keywords:** mTOR, neuroinflammation, alzheimer's disease, post translational modification, LPS

## Abstract

Neuroinflammation is a hallmark of Alzheimer's disease and TNFα as the main inducer of neuroinflammation has neurodegenerative but also pro-regenerative properties, however, the dose-dependent molecular changes on signaling pathway level are not fully understood. We performed quantitative proteomics and phospho-proteomics to target this point.

In HT22 cells, we found that TNFα reduced mitochondrial signaling and inhibited mTOR protein translation signaling but also led to induction of neuroprotective MAPK-CREB signaling. Stimulation of human neurons with TNFα revealed similar cellular mechanisms. Moreover, a number of synaptic plasticity-associated genes were altered in their expression profile including *CREB*.

SiRNA-mediated knockdown of CREB in human neurons prior to TNFα stimulation led to a reduced number of protein/phospho-protein hits compared to siRNA-mediated knockdown of CREB or TNFα stimulation alone and countermeasured the reduced CREB signaling. *In vivo* data of TNFα knockout mice showed that learning ability did not depend on TNFα per se but that TNFα was essential for preserving the learning ability after episodes of lipopolysaccharide-induced neuroinflammation. This may be based on modulation of CREB/CREB signaling as revealed by the *in vitro* / *in vivo* data.

Our data show that several molecular targets and signaling pathways induced by TNFα in neurons resemble those seen in Alzheimer's disease pathology.

## INTRODUCTION

Elevated levels of the cytokine tumor necrosis factor-α (TNFα) have been associated with neurodegenerative diseases such as Alzheimer's disease (AD) where increased levels of TNFα has been found in brains and cerebrospinal fluid of AD patients [1]. Systemic inflammation leading to increased TNFα is associated with disease progression/cognitive decline in AD patients [2], and systemic challenge, such as administration of lipopolysaccharide (LPS), can provoke waves of TNFα expression in the rodent brain [3]. The signaling events triggered by TNFα that culminate into neurodegeneration are still unclear but may include an imbalance in molecular pathways where TNFα contributes to neuronal injury but also exerts protective effects [4]. Accumulating knowledge from trials investigating anti-inflammatory drugs as candidates to prevent or slow progression of neurodegenerative diseases have also revealed controversial results [5]. Similarly, TNFα inhibitors have been used to eliminate TNFα in order to prohibit cell death in an AD phase 2 trial [6] but only with marginal effects. These observations may rely on the reductions of the neuroprotective properties of cytokines and suggests that a better understanding of the pathway dynamics is mandatory.

TNFα acts on the two receptors, TNFR1 and TNFR2, which are generally hold to have opposing effects e.g. on cell survival [7]. TNFR1, which contains a death domain, mainly shows neurodegeneration signaling although it also reveals neuroprotection *in vivo* [8] while TNFR2, which is without a death domain, is neuroprotective [9]. Importantly, it has been shown that local increase of TNFα in the hippocampal dentate gyrus activates astrocytic TNFR1, which in turn triggers an astrocyte-neuron signaling cascade that results in the persistent functional decline of hippocampal excitatory synapses [10]. In this context, TNFα exerts additional control of hippocampal synapses via α-amino-3-hydroxy-5-methyl-4-isoxazolepropionic acid (AMPA) [11] and gamma-aminobutyric acid (GABA) [12] receptor trafficking emerging as a key physiological regulator of hippocampal synaptic plasticity and modulator of neural injury [13]. Notably, it has been shown that TNFR1-triggered signaling might mediate mitochondrial function and induces apoptosis in neurons *in vitro* [13]. Neuroprotection via TNFR2 signaling may involve upregulation of BDNF protein, decrease in glutaminase levels and modulation of N-methyl-D-aspartate (NMDA) receptors [14]. Despite the fact that each receptor type mediates distinct cellular responses, there are also evidences of considerable overlap of their signaling capabilities in mediating biological outcomes [15–17]. Noteworthy, TNFR1-mediated apoptosis occurs during high TNFα levels whereas small amounts of TNFα in the low ng/ml range activate the TNFR2-mediated signaling cascade [7, 18].

We believe that the underlying molecular mechanisms and cellular responses of TNFα need to be investigated in a dose-dependent and global manner in order to better understand AD etiology and other neurological diseases where TNFα plays a pivotal role in triggering neuroinflammation and affecting synaptic plasticity.

## RESULTS

### Dose-dependent inhibition of cell proliferation and induction of apoptosis by TNFα in HT22 cells

**Figure 1:**
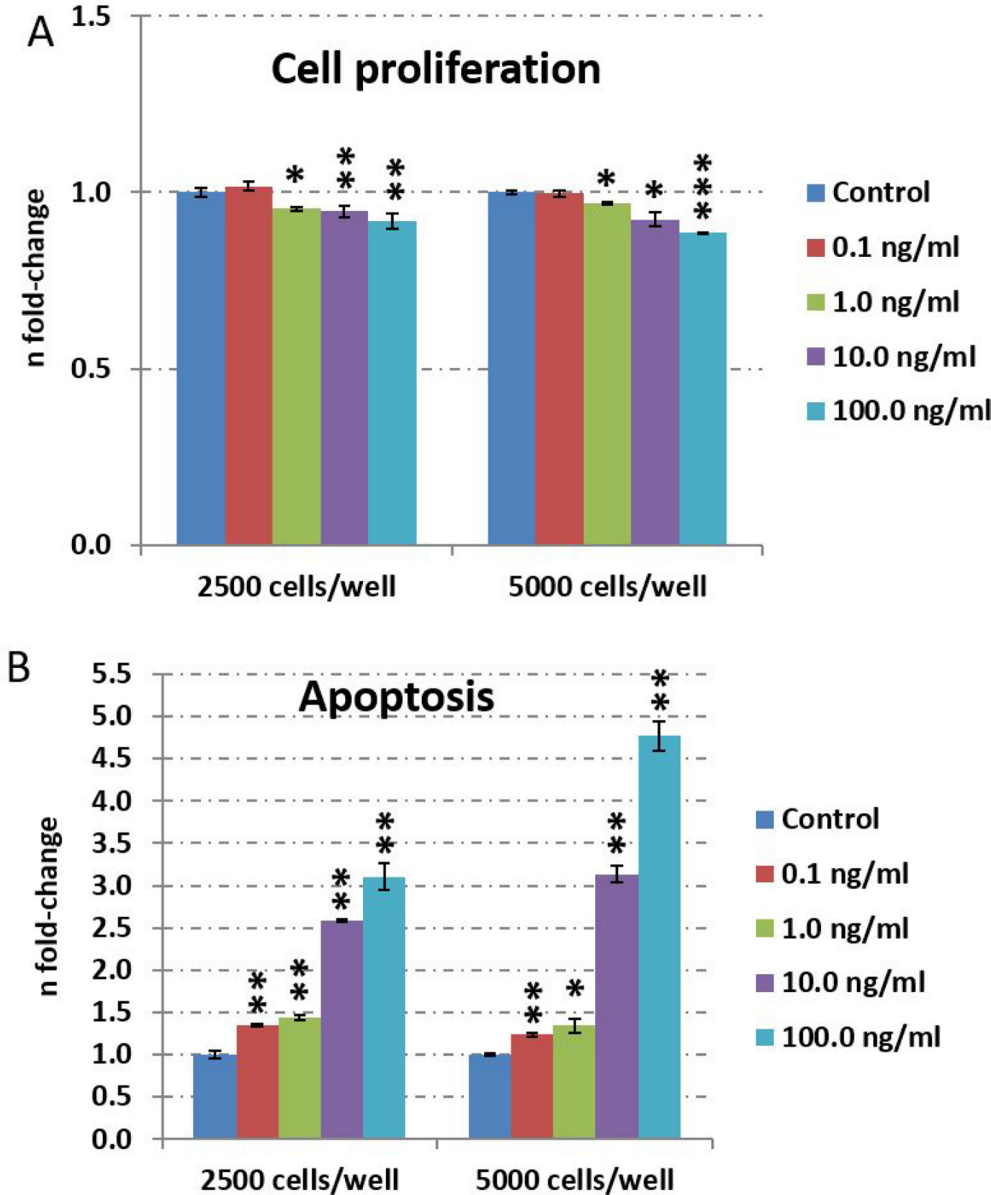
TNFα dose-dependent evaluation of cell proliferation and apoptosis in HT22 cells Cell proliferation (**A**) and apoptosis (**B**) was quantified by CellTiter-Glo^®^ Cell viability assay and Caspase-Glo^®^ 3/7 assay, respectively. Cells were cultured with a density of 2500 or 5000 per well in 96-well plates. The cells were stimulated with 0.1, 1, 10 or 100 ng/ml TNFα for 24 hours and untreated cells served as control. The figures show the fold-change and the standard error of the mean of five biological replicates per group. Statistical analysis was performed with unpaired Student's test; *p < 0.05; **p < 0.01; ***p < 0.001.

To investigate dose-dependency of TNFα on HT22 neuronal cells, we incubated 2500 cells/well and 5000 cells/well with increasing TNFα concentrations from 0.1 ng/ml to 100.0 ng/ml for 24 hours. We observed a dose-dependent inhibition of cell proliferation starting at 1.0 ng/ml whereas cell death was induced already at 0.1 ng/ml TNFα (Figure 1A and 1B). Based on these observations, we performed further experiments with a maximal dose of 10.0 ng/ml and excluded 100.0 ng/ml as it may over-induce apoptosis signaling (Figure 1B).

### Global mass spectrometry-based protein and phospho-protein quantification reveals dose-dependent impairment in energy metabolism and synaptic plasticity in HT22 cells

**Figure 2:**
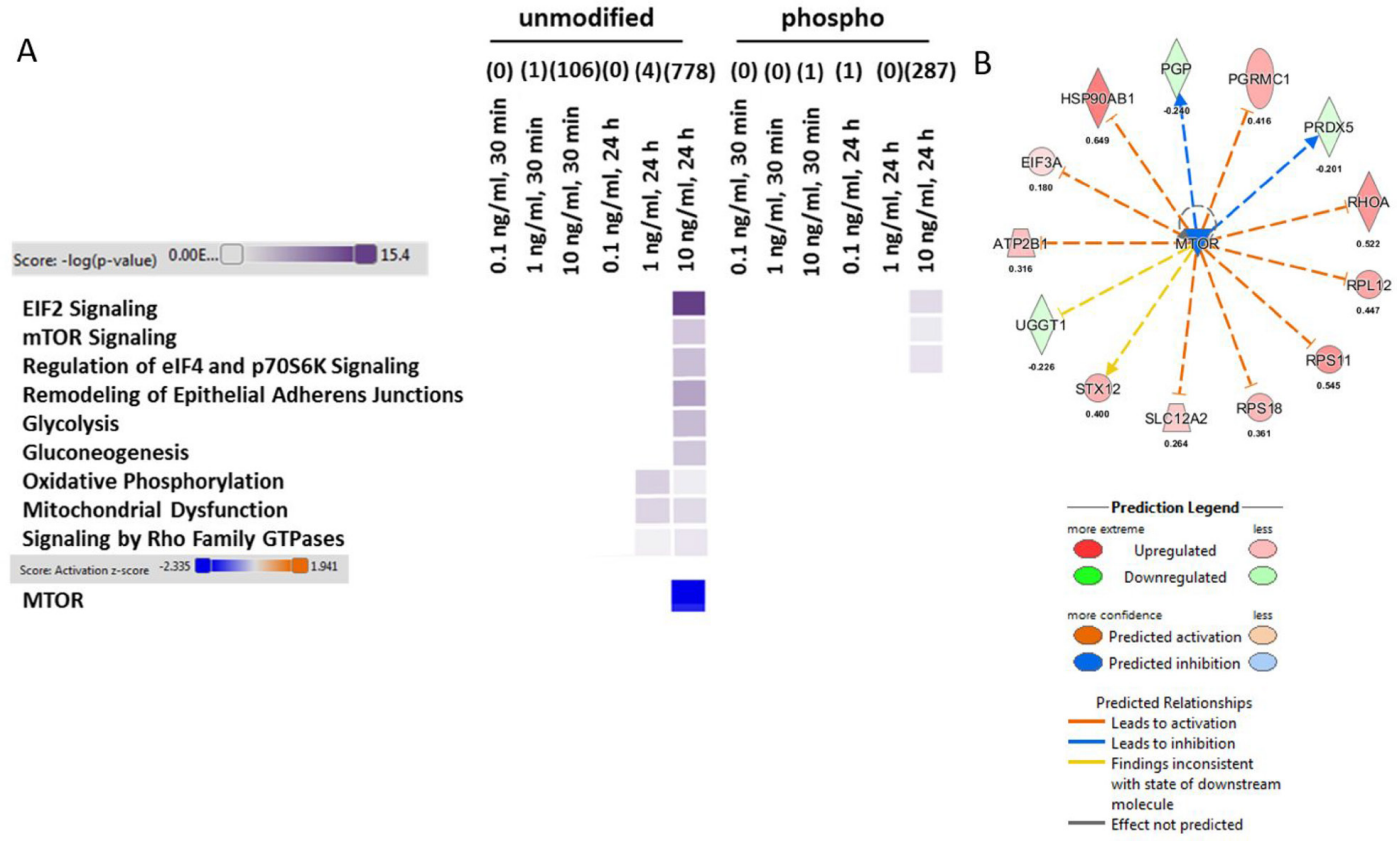
Analysis of signaling pathways from proteomics and phospho-proteomics experiments in HT22 cells Altered TNFα dose-dependent signaling pathways in HT22 cells on the proteome and phospho-proteome using Ingenuity Pathway Analysis software are shown in panel (**A**) High colour intensity represents high significance (*p* value) of the pathway. All coloured boxes have a *p* value ≤ 0.05; white boxes have a *p* value ≥ 0.05 and are not significantly changed; *n* = 3 in each group. Panel (**A** and **B**) show the upstream regulator analysis from IPA software (z-score > 2.0: predicted significant activation of node; z-score < 2.0: predicted significant inhibition of node) were grouped and visualized from proteins (unmodified) of 10 ng/ml TNFα stimulation over 24 hours. There was no significant change in the other groups.

To elucidate the molecular mechanism behind low dose (0.1 and 1.0 ng/ml) and moderate TNFα concentrations (10.0 ng/ml), we performed a global quantitative proteome and phospho-proteome analysis after 30 minutes and 24 hours of TNFα stimulation in HT22 cells. We noted that the number of significantly changed proteins (0.1 ng/ml/1 ng/ml: 0/1 (30 minutes) and 0/4 (24 hours)) and phospho-proteins (0.1 ng/ml/1 ng/ml: 0/0 (30 minutes) and 1/0 (24 hours)) induced by the two lower TNFα concentrations are scarce compared with the high numbers of alterations seen at 10.0 ng/ml dose (proteins: 106 (30 minutes) and 778 (24 hours); phospho-proteins: 1 (30 minutes) and 287 (24 hours)) (Figure 2A, Supplementary Table 5). Supplementary Tables 1–4 show the identifiable and quantifiable protein and phospho-protein hits.

Bioinformatics analysis of signaling pathways using IPA software showed that energy production (Glycolysis, Gluconeogenesis, Oxidative Phosphorylation and Mitochondrial Dysfunction) and cell proliferative / protein-translational signaling pathways (Eif2 Signaling, mTOR Signaling, Regulation of eiF4 and p70S6K Signaling) as well as synaptic plasticity-associated signaling (Signaling by Rho Family GTPases and Remodeling of Adherens Junctions) were not changed at the 30 minutes time point either at protein or phospho-protein level (Figure 2A). However, we noted that the affected signaling pathways were all significantly modulated by a TNFα dose of 10 ng/ml after 24 hours of incubation on the protein level whereas only EIF2 Signaling, mTOR Signaling and Regulation of eif4 and p70S6K Signaling correlated in addition on phospho-level to the pathways changes seen on protein level (Figure 2A). Notably, we observed a significant inhibition of mTOR at 10.0 ng/ml TNFα stimulation for 24 hours (Figure 2A and 2B) based on our deregulated proteins (Supplementary Table 5)

### TNFα-stimulation of HT22 cells leads to reduction in mitochondrial protein expression and activation of synaptic plasticity-associated CREB signaling

**Figure 3:**
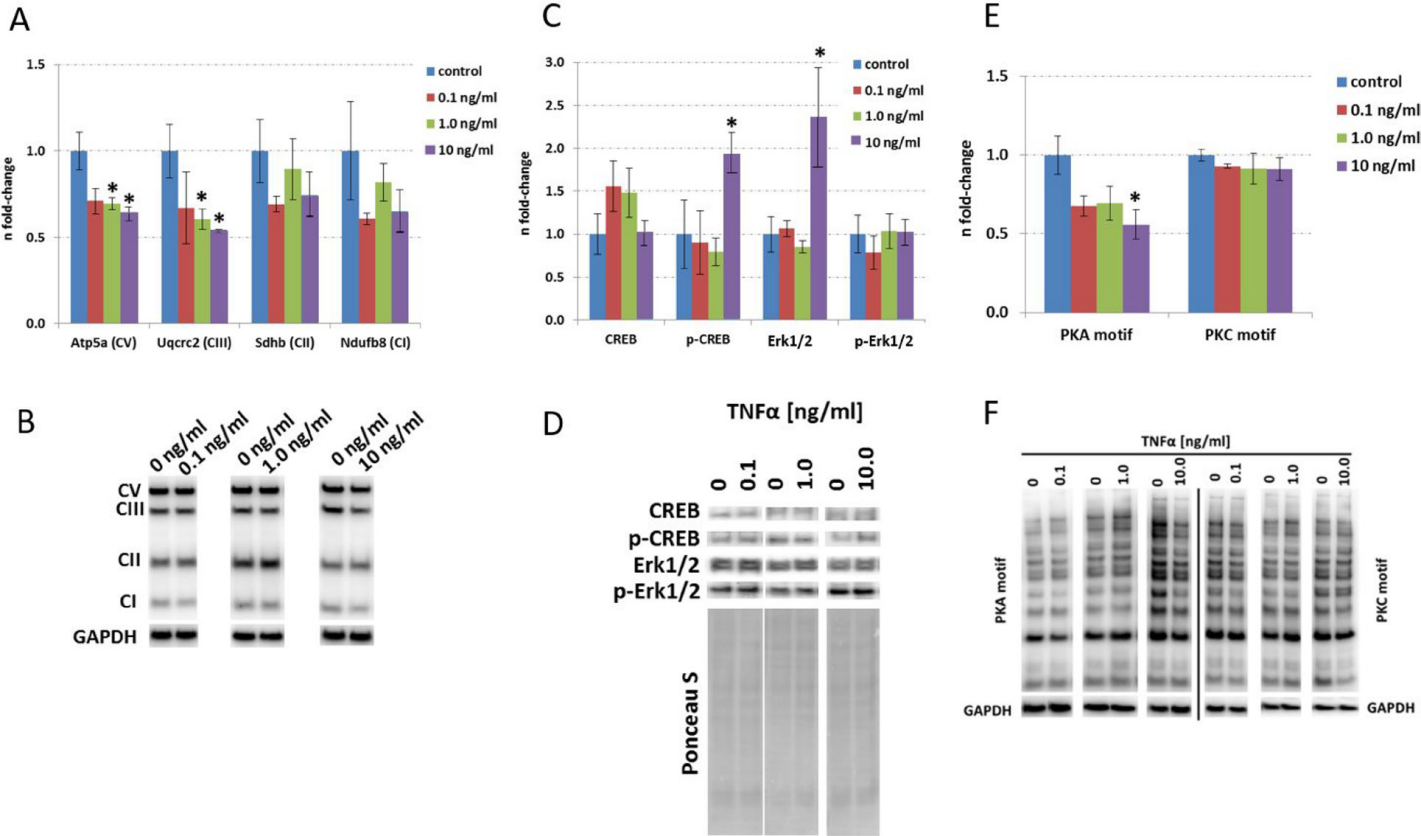
Immunoblotting of targets involved in mitochondrial function, synaptic plasticity and mTOR signaling in HT22 cells Data from immunoblots of mitochondrial protein subunit expression (**A**), CREB, p-CREB, MAPK and p-MAPK (**C**) as well as PKA and PKC motif immunoblotting (**E**) are depicted from 0 ng/ml, 0.1 ng/ml, 1.0 ng/ml and 10.0 ng/ml TNFα stimulation over 24 hours in HT22 cells. The columns represent the fold-changes with standard errors of the mean [67]; *n* = 3; *p < 0.05; **p < 0.01; ***p < 0.001 (unpaired Student s *t*-test). Normalization was performed against endogenous GAPDH for mitochondrial proteins as well as PKA and PKC motif immunoblotting; CREB, p-CREB, MAPK and p-MAPK were normalized against total lane intensity via Ponceau S staining. Representative visualisation of the immunoblotting data is shown in Panel **B**, **D** and **F**.

To validate the TNFα-induced changes in signaling pathways, we performed immunoblotting of key proteins associated within these signaling pathways. The mitochondrial proteins Atp5a (Complex V) and Uqcrc2 (Complex III) but not Sdhb (Complex II) or Ndufb8 (Complex I) were significantly reduced in their expression levels after 24 hours of TNFα incubation only at a dose of 1.0 and 10.0 ng/ml (Figure 3A and 3B) correlating well with the bioinformatics-based deregulated signaling pathways of Oxidative Phosphorylation and Mitochondrial Dysfunction at these doses (Figure 2A). Proteomics revealed an increased protein expression of Mtco2 and Cox3 (Complex IV) at 1.0 ng/ml as well as increased protein expression of Atp5l (Complex V) and a number of abundance alterations of Complex I proteins (decrease in Ndufs7, Ndufs8, Ndufc2; increase in Ndufs2) at 10.0 ng/ml 24 hours TNFα stimulation for 24 hours (Supplementary Table 5). Sdhb (Complex II) was significantly increased in the proteomics data set (Supplementary Table 5) whereas immunoblotting showed no change (Figure 3A and 3B).

Next, we quantified CREB and p-CREB levels as they are essential synaptic plasticity regulators within neurons and modulate Rho signaling [19] which we noted to be affected in HT22 neurons by TNFα (Figure 2A). We observed that p-CREB but not CREB levels were significantly increased at a dose of 10.0 ng/ml TNFα whereas their pattern was not changed at lower doses (Figure 3C and 3D).

To validate our observations in cell proliferative / protein translational signaling pathways, we quantified MAPK (Erk1/2), p-MAPK (p-Erk1/2) as well as total phospho-proteins with PKA and PKC phosphorylation consensus motif as surrogates for these pathways as mTOR and MAPK pathways converge on eIF4 signaling [20]. We only noted an increase in MAPK but not p-MAPK levels at a dose of 10.0 ng/ml TNFα (Figure 3C and 3D) that was accompanied by a decrease in total PKA upstream of MAPK signaling but not PKC activity as revealed by phospho-PKA/-PKC Substrate Antibody immunoblotting (Figure 3E and 3F).

### Increased cell proliferation potential by low-dose TNFα stimulation in human neurons

Next, we changed our *in vitro* model to human embryonic stem cell-derived neurons to elucidate the molecular neuronal mechanism of TNFα closer to human neuronal physiology. By using immunofluorescence, we show that the differentiation process resulted in a neuronal population (β-tub III staining) with very few Ki67-positive proliferating NSCs and a substantial number of GABAergic neurons (Supplementary Figure 1). Moreover, very few GFAP-positive astrocytes were observed in the cultures (not shown).

**Figure 4:**
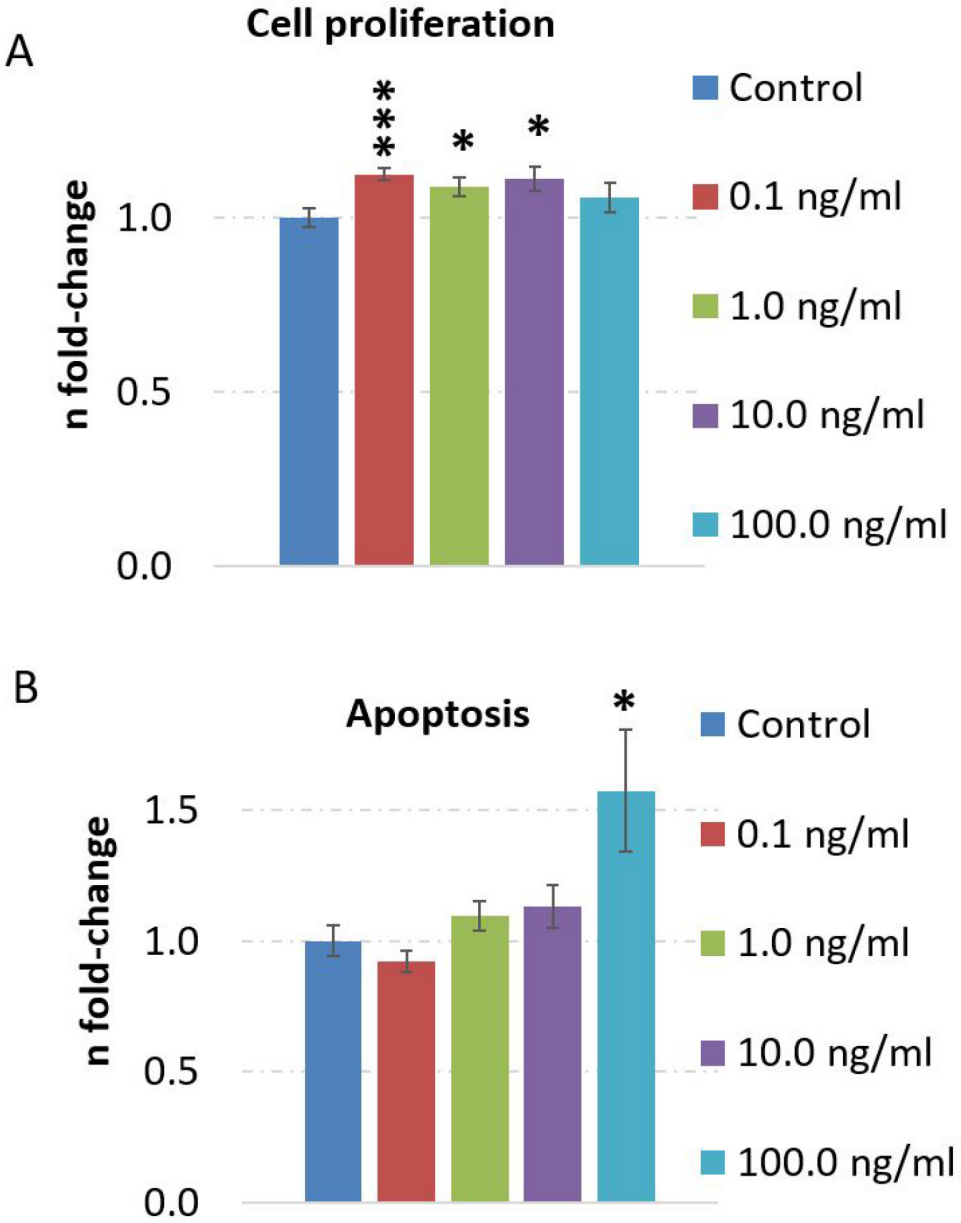
TNFα dose-dependent evaluation of cell proliferation and apoptosis in human neurons Cell proliferation (**A**) and apoptosis (**B**) was quantified by CellTiter-Glo^®^ Cell viability assay and Caspase-Glo^®^ 3/7 assay, respectively. Cells were cultured with a density of 7000 cells per well (96-well plates). The cells were stimulated with 0.1, 1, 10 or 100 ng/ml TNFα over 24 hours and untreated cells served as control. The figures show the fold-change and the standard error of the mean of 7-8 biological replicates per group. Statistical analysis was performed with unpaired Student's test; *p < 0.05; **p < 0.01; ***p < 0.001.

We also investigated the dose-dependency of TNFα and its influence on cell death and cell proliferation. We noted that apoptosis is significantly induced by 100.0 ng/ml TNFα stimulation over 24 hours but not at lower doses (Figure 4B) whereas cell proliferation was increased at 0.1 ng/ml, 1.0 ng/ml and 10.0 ng/ml dose but not at 100.0 ng/ml dose (Figure 4A) in human neurons.

### TNFα influences signaling pathways of mTOR, protein synthesis and mitochondrial function in human neurons

**Figure 5:**
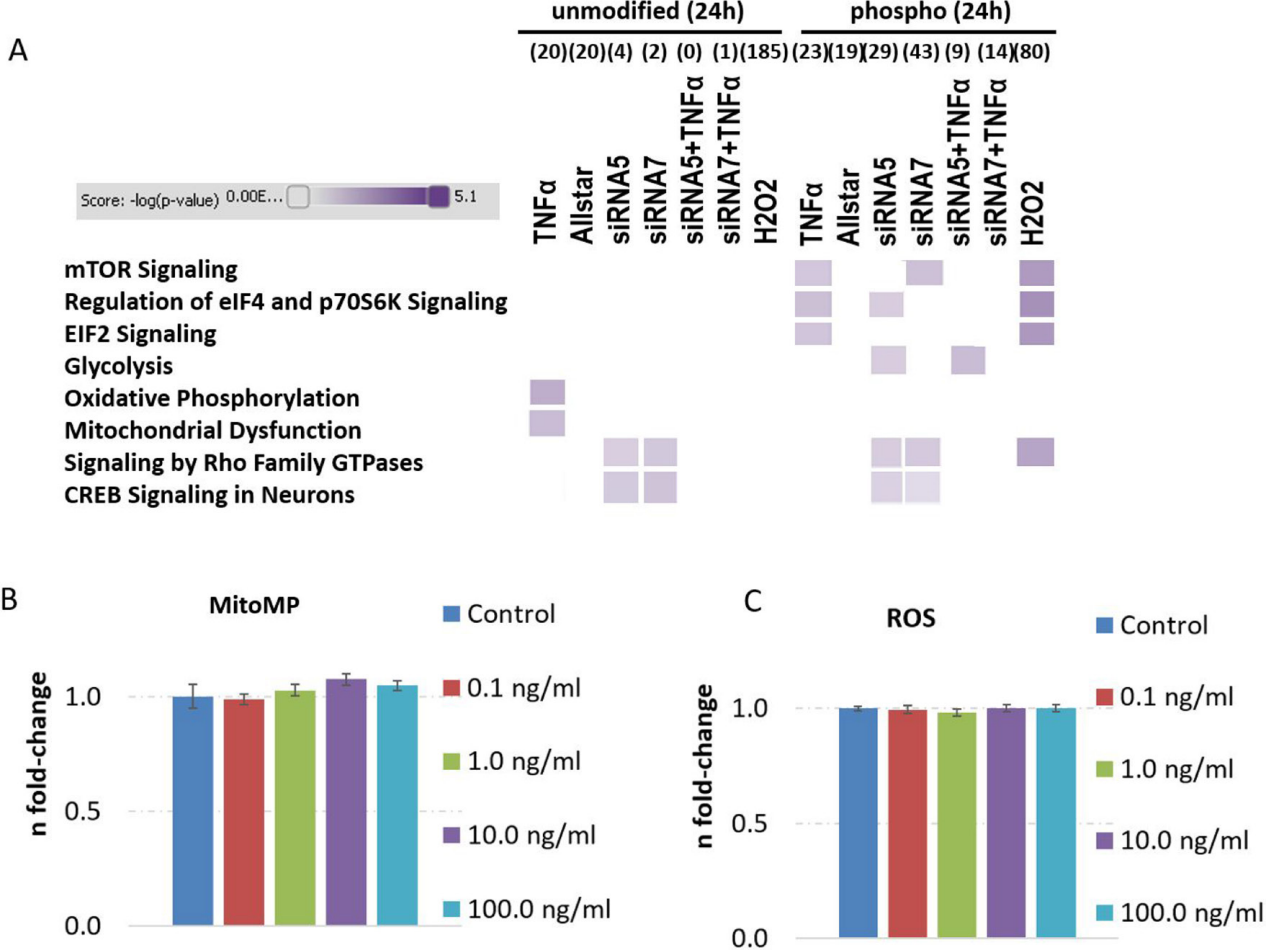
Analysis of signaling pathways from proteomics and phospho-proteomics experiments and quantification of mitochondrial membrane potential as well as reactive oxygen species levels in human neurons treated with TNFα Altered signaling pathways in human neurons on the proteome and phospho-proteome using Ingenuity Pathway Analysis software are shown in panel (**A**) with the following treatment groups over 24 hours: TNFα-dose of 1 ng/ml alone, Allstar as transfection control, siRNA5 and siRNA7 against CREB expression, siRNA5 and siRNA7 knockdown prior to 24 hours TNFα (1 ng/ml) incubation and H_2_O_2_ incubation serving as positive control. High colour intensity represents high significance (*p* value) of the pathway. All coloured boxes have a *p* value ≤ 0.05; white boxes have a *p* value ≥ 0.05 and are not significantly changed; *n* = 2 in each group. Panel (**B** and **C**) show mitochondrial membrane potential (MitoMP) and reactive oxygen species (ROS) quantification from 7000 cells per well showing the fold-change and the standard error of the mean of 7–8 biological replicates per group. Statistical analysis was performed with unpaired Student's test; *p < 0.05; **p < 0.01; ***p < 0.001.

Global mass spectrometry-based proteomics and phospho-proteomics of 1.0 ng/ml TNFα stimulation of human neurons over 24 hours showed a deregulation of 20 proteins and 23 phospho-proteins (Figure 5A, Supplementary Table 10). Supplementary Tables 6–9 show the identifiable and quantifiable proteins and phospho-proteins. Bioinformatics analysis of signaling pathways using IPA software showed changes in mitochondrial function (Oxidative Phosphorylation, Mitochondrial Dysfunction) and cell proliferative/protein-translational signaling pathways (Eif2 Signaling, mTOR Signaling, Regulation of eiF4 and p70S6K Signaling) (Figure 5A).

Altered mitochondrial function was validated by a MitoMP assay but did not show any dose-dependent TNFα differences (Figure 5B) although proteomics data indicate an increase in protein subunits of CIV (COX5A) and CV (ATP5O) (Supplementary Table 10). Moreover, ROS levels were also unchanged by dose-dependent TNFα incubation in human neurons over 24 hours (Figure 5C). Thus, alterations in ROS signaling seems to be not attributable for apoptosis induction (Figure 4B), cell proliferation elevation (Figure 4A) and -signaling (Figure 5A).

### SiRNA-mediated knockdown of CREB prior to TNFα stimulation leads to less changed proteins and phospho-proteins compared to knockdown or TNFα treatment alone in human neurons

CREB is a transcription factor connecting a multitude of pathways related to neuronal metabolism/proliferation, synaptic plasticity and mitochondrial function [21], that were affected targets in our HT22 cell study (Figures 1, 2, 3) accompanied by elevated levels of p-CREB (Figure 3C and 3D). Therefore, we performed a siRNA-mediated knockdown of CREB protein in human neurons prior to TNFα treatment to investigate the CREB/CREB signaling contribution toward the molecular mechanism of TNFα. The siRNA treatment resulted in an average CREB knockdown efficiency of 50% (siRNA5: 48% and siRNA7: 53% compared to all-star negative controls; *n* = 2) as shown by immunoblotting (Supplementary Figure 2).

Controls (negative – Allstar transfection; positive – H_2_O_2_ stimulation) revealed no changes in signaling pathways both at protein (Allstar and H_2_O_2_) and phospho-protein level (Allstar) (Figure 5A) whereas 20/185 proteins (Allstar/H_2_O_2_) and 19/80 phospho-proteins (Allstar/H_2_O_2_) were altered in their expression profile (Supplementary Table 10). Supplementary Tables 6–9 show the identifiable and quantifiable proteins and phospho-proteins. Only mTOR Signaling, Regulation of eIF4 and p70S6K Signaling, EIF2 Signaling and Signaling by Rho Family GTPases were deregulated on the phosphorylation status by H_2_O_2_ stimulation (Figure 5A).

SiRNA-mediated knockdown of CREB using two independent siRNAs approaches (siRNA5 and siRNA7) affected CREB Signaling in Neurons and Signaling by Rho Family GTPases on the proteome and phospho-proteome level (siRNA5/siRNA7: proteome 4/2; phospho-proteome 29/43) (Figure 5A). Rho GTPases such as Rac1 and Cdc42 playing a fundamental role of CREB activity regulation [22–24]. CREB protein expression was significantly down-regulated as observed by immunoblotting (Supplementary Figure 2) whereas proteomics showed a tendency of downregulation (Supplementary Table 11). Further signaling pathways on the phospho-proteome level were affected by siRNAs but not by both simultaneously (Figure 5A) indicating only random hits. Most proteins and phospho-proteins overlap within the two siRNA knockdown approaches (Supplementary Table 10 – highlighted in green). Importantly, siRNA-mediated CREB knockdown prior to 1.0 ng/ml TNFα stimulation does neither alter CREB signaling nor Rho Family GTPase signaling with 0/1 altered proteins and 9/14 phospho-proteins for siRNA5+TNFα and siRNA7+TNFα, respectively (Figure 5A, Supplementary Table 10). Immunoblotting of CREB demonstrated that the CREB protein levels were not changed by 1.0 ng/ml TNFα whereas siRNAs and H_2_O_2_ stimulation significantly reduces expression levels (Supplementary Figure 2). Importantly, Allstar and siRNAs+TNFα did not affect CREB expression (Supplementary Figure 2) correlating with our pathway analysis results (Figure 5A).

### Gene expression analysis reveals impairment in synaptic plasticity by TNFα stimulation in human neurons

**Figure 6:**
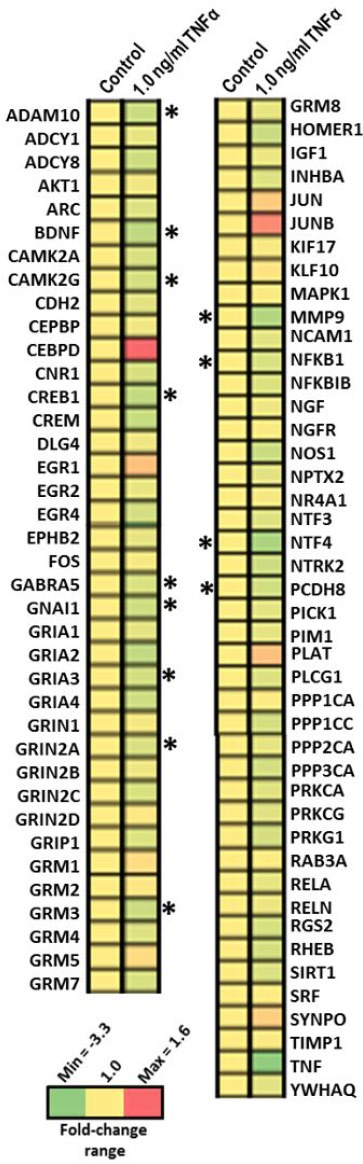
Quantification of the expression of genes involved in synaptic plasticity after 1.0 ng/ml TNFα stimulation over 24 hours in human neurons Genes significantly changed in expression after 1.0 ng/ml TNFα stimulation for 24 hours compared to untreated human neurons using RT2 Profiler PCR Arrays (Qiagen) are shown. The table shows the genes which are significantly up-regulated or down-regulated compared to controls in a heat-map presentation; *p < 0.05; **p < 0.01; ***p < 0.001 (unpaired Student's *t*-test, *n* = 3).

Next, we quantified 84 mRNAs related to synaptic plasticity to investigate the molecular mechanism of TNFα in more detail. Thirteen gene transcripts were significantly down-regulated in their expression profile in human neurons treated with 1.0 ng/ml TNFα for 24 hours (Figure 6, Supplementary Table 12) including neuronal receptors (GABRA5, GRIA3, GRIN2A, GRM3), neurotrophic factors (BDNF, NTF4), cell-matrix modulators (ADAM10, MMP9, PCDH8), cellular stress responder (NFKB1) and neurotransmission regulators (CAMK2G, CREB1, GNAI1).

### TNF knockout mice have learning- and memory-related cognitive deficits after lipopolysaccharide-induced inflammation

**Figure 7:**
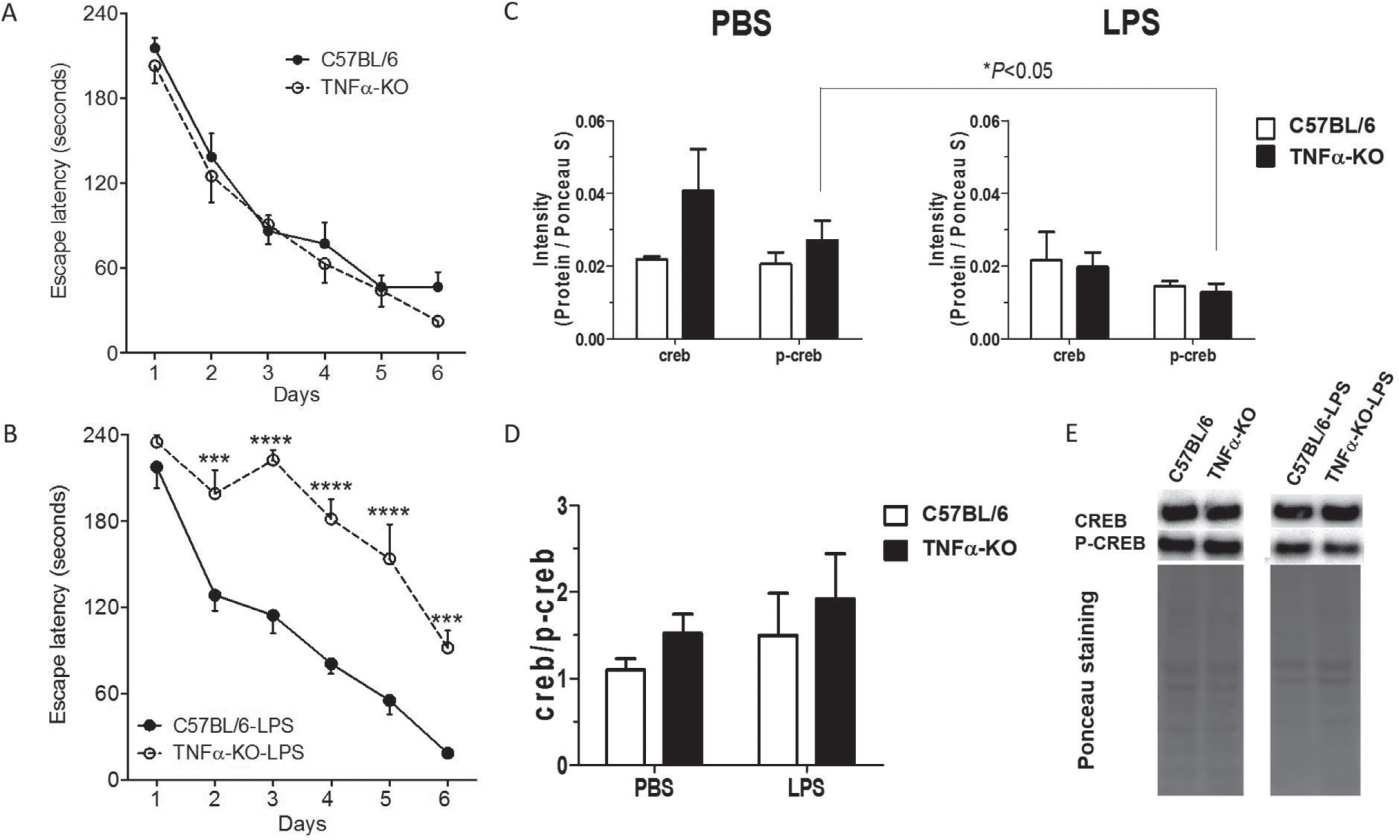
Neuroinflammation induced by LPS in TNFα knockout mice impairs cognitive ability (**A** and **B**) Hippocampus-dependent cognitive ability was explored in the Barnes maze in unmanipulated (A) and LPS-treated (B) female C57BL/6 (filled circles) and TNFα-KO (open circles) mice. The escape latency represents the group mean of the average latency for 4 trials per mouse during the 6 days test period. Mice in (B) received an intraperitoneal injection of LPS (0.5 mg/kg of serum E-coli 0111:B4), 7 and 4 days prior to the Barnes maze learning experience. Error bars represent standard error of the mean [67]. (A) C57BL/6 mice (*n* = 16) and TNFα-KO mice (*n* = 11) showed similar learning ability during the acquisition phase. Two-way ANOVA, repeated measures: Interaction, F(5,125) = 0.45, *P* = 0.0010; Group, F(1,25) = 0.81, *P* = 0,3779; Time, F(5,125) = 74,36, *P* < 0,0001; Matching, F(5,125) = 74.36, *P* <= 0,0001. Sidak's multiple comparison test: ns, for all comparisons. (B) In comparison, LPS injection seriously impaired the learning ability of the TNFα-KO mice (*n* = 8), but not in the C57BL/6 mice (*n* = 8). LPS-injected TNFα-KO mice consistently showed a significant higher latency in locating the goal box. Two-way ANOVA, repeated measures: Interaction, F(5,70) = 4.68, *P* = 0.0010; Group, F(1,14) = 50.85, *P* < 0,0001; Time, F(5,70) = 59,23, *P* < 0,0001; Matching, F(14,70) = 3.03, *P* = 0,0011. Sidak's multiple comparison test, ***P < 0,001; ****P < 0,0001. KO; knockout, ns; not significant, LPS; lipopolysaccharide. (**C**–**E**) Immunoblotting of CREB and p-CREB of hippocampus from three-month-old C57BL/6 and TNFα knockout mice (male and female) to explore the connection between TNFα and CREB signaling under physiological and inflammatory conditions. Mice were treated with a single intraperitoneal injection of PBS or LPS (0.5 mg/kg of serum E-coli 0111:B4) in sterile PBS and were sacrificed four hours after the injection. Intensities of protein CREB and p-CREB were normalised against respective total lane intensity of Ponceau staining. Statistical analysis was performed by using unpaired Student's *t*-test (*n* = 3–6/treatment group & genotype, total number of mice: *n* = 17). Bars represent the mean±SEM; Representative images of CREB and p-CREB immunoblotting and Ponceau stainings are shown in panel E.

Our *in vitro* data indicated that TNFα may be important in the memory-related CREB signaling regulation in neurons. Thus, we questioned if knockout of TNFα in mice affects hippocampal-dependent learning and memory-related behaviour, which we did by exposing TNFα knockout and age-matched C57BL/6 mice to the Barnes maze. Both groups of mice quickly learned to locate the goal box and the latency times of both groups were significantly decreased from day 2 of the 6 days the mice were tested, with no difference between the groups (Figure 7A), indicating that TNFα is not required for learning under normal conditions. Next, the TNFα knockout and C57BL/6 mice were challenged by inducing an inflammatory reaction by intraperitoneal LPS injection on days 7 and 4 prior to learning. The time of the injections was guided by observations obtained in the Morris water maze, showing that water maze-induced learning promotes the survival of the granule cell precursors born a few days prior to induction of the learning experience [25]. As expected, LPS-injected mice displayed sickness behaviour that ceased 2–3 days after the last LPS-injection, which was 1–2 days prior to the time point where the mice were exposed to the Barnes maze protocol (taking place 4 days after the last LPS-injection). Interestingly, the LPS-injected TNFα knockout mice were extremely slow learners compared to the LPS-injected C57/BL6 mice (Figure 7B). Comparative analysis of toluidine blue stained coronal brain sections revealed no histoarchitectonic abnormalities in LPS-injected TNFα knockout compared to LPS-injected C57/BL6 mice. In particular, the hippocampus, which is involved in spatial learning and memory, had completely normal appearing granule and pyramidal cell layers and normal appearing neuropil layers along the entire rostrocaudal axis (data not shown). No changes in weight of the LPS-injected TNFα knockout or C57BL/6 mice were observed over the experiment (data not shown).

To explore the connection between TNFα and CREB signaling under physiological and LPS-induced inflammatory conditions, corresponding to those induced at 7 or 4 days prior to the Barnes maze-induced learning experience, we next challenged three-month-old C57BL/6 and TNFα knockout mice to a single intraperitoneal injection with either LPS (0.5 mg/kg) or vehicle PBS and sacrificed them 4 hours after the injections corresponding to the time when inflammation peaks in the brain after LPS-injection [26]. Subsequently, the hippocampus was isolated and CREB, p-CREB immunoblotting was performed. Figure 7C–7E show a significant reduction of p-CREB levels in LPS-treated versus PBS-treated TNF-KO mice in the hippocampus whereas CREB/p-CREB ratios were unchanged between C57BL/6 and TNF-KO regardless of PBS and LPS-stimulation.

These data demonstrate that acute LPS-induced inflammation prior to learning affects hippocampus-dependent learning ability in TNFα knockout mice whereas modulation of CREB signaling seems to be an important molecular target in the LPS response.

## DISCUSSION

To characterise neurodegenerative and pro-neurogenic properties of TNFα, we used quantitative proteomics and phospho-proteomics in HT22 neuronal cells and human embryonic stem cell-derived neurons to elucidate the differences in signaling pathway profiles that are associated with the distinct molecular properties of TNFα. Furthermore, we performed experiments with siRNA-mediated CREB knockdown and behavioral learning analysis of TNFα knockout mice compared to wild type mice performance challenged with LPS.

HT22 cells are suitable as neuronal model to study TNFα signaling since they express both receptor subtypes for TNFα (TNFR1 and TNFR2) under normal culture conditions [13]. It has previously been shown that HT22 cells treated with TNFα at concentrations of 1.0 and 10.0 ng/ml for 4 hours lead to up-regulation of *Homer1a* and *Egr2* [27], whereas both molecules are important in synaptic plasticity and memory formation. These data correlate well with our noted activation of CREB-mediated signaling at a dose of 10.0 ng/ml over 24 hours as *Homer1a* [28] and *Egr2* [29] are CREB-dependent genes. As MAPK signaling is an upstream regulator of CREB signaling, the increase in MAPK at a dose of 10.0 ng/ml TNFα correlates with potential higher CREB activity. Scientific data indicate that an elevated MAPK signaling is involved in early stages of AD [30–32]. Importantly, the increased MAPK signaling in AD occurs via cellular stress arising from different factors such as TNFα [33].

It has been reported that pretreatment (24 hours) of murine hippocampal slice cultures with 10.0 ng/mL TNFα potentiated AMPA-induced neuronal death [18]. Moreover, Doll et al. noted that TNFα stimulation of HT22 cells decreased the cell viability. They also demonstrated that TNFα exerts its neurotoxic effects through TNFR1 but not TNFR2 accompanied with apoptosis induction and decreased mitochondrial membrane potential [13]. We also observed a dose-dependent alleviation in cell proliferation and induction of apoptosis by TNFα in HT22 cells. Importantly, only a dose of 10.0 ng/ml TNFα led to significant alterations of signaling pathways associated to cell metabolism, protein translation, mitochondrial function and synaptic plasticity. Notably, we found that mTOR, an important protein in regulating all these biological targets, is inhibited as revealed by bioinformatic analysis of proteome data. It has been shown that inhibition of mTOR is beneficial for synapse-dependent neuroprotection in transgenic AD mouse models reducing the levels of amyloid beta [34] and abrogating tau-mediated neurotoxicity by using systemic rapamycin [35]. Noteworthy, inhibition of mTOR signaling seems to be inversely correlated with TNFα expression in an AD mouse model (APP_swe_PS1_dE9_) compared to wildtype control mice within the neocortex and hippocampus [36]. The elevation of synaptic plasticity-regulating and neuroprotective-acting p-CREB levels correlate well with the biological down-stream effects of indicated mTOR inhibition in HT22 cells at a dose of 10.0 ng/ml TNFα in our study. Furthermore, the reduction in PKA activity as revealed by motif-immunoblotting is also in agreement with mTOR inhibition as the pharmacological elevation of cAMP – an upstream inducer of PKA signaling - in mouse embryonic fibroblasts and HEK293 cells inhibits mTORC1 activation via a PKA-dependent mechanism [37].

Moreover, it has been reported that the bioenergetic effect of mTOR inhibition in neurons significantly preserves neuronal ATP levels, particularly when oxidative phosphorylation is impaired [38]. Our data indicated that oxidative phosphorylation is affected by TNFα stimulation in HT22 cells influencing protein abundances of Complex I, -III, -IV and -V subunits. As the mitochondrial antibody cocktail used for immunoblotting consists only of the core proteins important for the assembly of the mitochondrial complex, the discrepancy between proteomics and immunoblotting data, particularly in the case of Complex V, is not unexpected and has been observed before [39, 40]. Neurons have a high ATP demand [41], and a reduction in ATP levels lead to profound decrease in neuronal viability [42]. Using a dopaminergic neuronal cell line (SH-SY5Y), it was also shown that TNFα alters Complex I activity, decreases ATP levels and increases ROS levels and mitochondrial turnover [43]. In contrary, we did not observe any changes in ROS level or MitoMP in human neurons treated with TNFα, which was seen in HT22 cells by Doll et al. [13]. We believe that this is due to the intrinsic molecular and cellular differences of the used neuronal cell lines (SH-SY5Y and HT22) in comparison to our human stem cell-derived neurons.

The increase in cell proliferation that we observed at 0.1, 1.0 and 10.0 ng/ml TNFα in human neurons has also been found by others. Bernardino et al. demonstrated that the exposure of murine subventricular neuronal cultures to 1.0 and 10.0 ng/ml mouse or 1.0 ng/ml human recombinant TNFα resulted in increased differentiation of cells displaying a neuronal-like calcium-mediated profile of responses, compared with the predominant profile of immature cells observed in non-treated control cultures [44]. Moreover, by using neutralizing antibodies for each TNFα receptor, they found that the pro-neurogenic effect of 1.0 ng/ml TNFα is mediated via TNFR1 activation. Interestingly, exposure of these cultures to 1.0 ng/ml TNFα induced cell proliferation, whereas 10.0 and 100.0 ng/ml TNFα induced apoptotic cell death [44]. The pro-survival molecular mechanism at 1.0 ng/ml TNFα stimulation in human neurons of our study may be attributable by decreased expression of p-BCLAF1 (Bcl-2-associated transcription factor 1) at Ser658 that is a positive regulator of apoptosis.

Noteworthy, our mRNA analysis revealed changes in several synaptic plasticity-regulating genes particular in expression levels of neuroreceptors in human neurons with 1.0 ng/ml TNFα stimulation over 24 hours. There, we noted that *CREB* was significantly down-regulated whereas CREB levels were unchanged by immunoblotting and proteomics experiments. CREB knockdown in human neurons led to changes in CREB-mediated signaling (Signaling of Rho family GTPases and CREB Signaling in Neurons) as expected. In comparison, CREB knockdown followed by TNFα stimulation indicated a balance of CREB signaling as revealed by immunoblotting and signaling pathway analysis of proteomic and phospho-proteomic data. These data indicate that TNFα and CREB signaling are interconnected in neurons that could be based on the cAMP-responsive element (CRE) that TNFα poses as reviewed elsewhere [45]. Thus, TNFα can modulate the signaling processes in neurons as well as learning and memory response to LPS-induced neuroinflammation as we demonstrated in our hippocampus-dependent behavioural testing.

To investigate how TNF affects learning ability, we exposed groups of TNFα knockout and age-matched C57BL/6 mice to the Barnes maze. We did not observe any learning deficits in TNFα knockout mice using our Barnes maze protocol. This is in line with previous reports in TNFα knockout mice where no learning impairments are observed during the acquisition phase [46, 47]. We challenged the TNFα knockout mice and age-matched C57BL/6 mice with LPS-induced inflammation prior to learning. We observed clear learning impairments, after sickness behaviour had ceased, in the TNFα knockout mice, indicating that TNFα plays a homeostatic role during neuroinflammation as has previously been suggested by others [48]. We also expected to find learning impairments in LPS-injected C57BL/6 mice, since LPS has been reported to impact on neurogenesis [49–51] and induce apoptosis of neurons in the subgranular zone [52]. However, we found that inflammation prior to learning essentially did not affect C57BL/6 mice compared to age-matched C57BL/6 mice. Others have shown learning deficits after LPS-injections in adult C57BL/6 mice using the Morris Water maze, yet, LPS injections were given 4 hours prior to learning [53] and the mice probably displayed sickness behaviour, which affects locomotor activity [26]. We did, however, find a slight learning impairment in LPS-treated 9-month-old C57BL/6 mice, in line with studies showing that aging causes a greater inflammatory response [54], which could cause greater impairments in learning ability. Importantly, we demonstrated that LPS- but not PBS-stimulation reduces p-CREB levels in TNFα-KO mice but not in WT mice in the hippocampus 4 hours after stimulation. Phosphorylated CREB is the active form and is implicated in the regulation of development, protection, learning, memory and plasticity in the nerve system [55]. Thus, ameliorating the levels of p-CREB in the hippocampus affect CREB signaling that may explain the reduced memory and learning performance in our hippocampus-dependent Barnes maze experiment. Whether or not these effects might also influence hippocampal neurogenesis, as might be inferred by studies by others [25], remains to be determined.

Our data suggest that the learning ability does not depend on TNFα but that TNFα preserves the learning and memory ability during episodes of neuroinflammation resembling the persistent neuroinflammation observed in AD. This may be based on modulation of CREB as revealed by our *in vitro* and *in vivo* studies, however, a deeper molecular analysis is necessary to clarify this connection in our model.

## MATERIALS AND METHODS

### Cultivation of neuronal HT22 cells and human neurons

HT22 cells (immortalised primary neurons from mouse hippocampus) were originally kindly provided by J. Lewerenz (Department of Neurology, University Hospital Hamburg-Eppendorf, Hamburg, Germany) [56] and were maintained in proliferation state in the laboratory of S. Tapio (Institute of Radiation Biology, Helmholtz Zentrum München, Munich, Germany) from where we received a frozen aliquot. The cells were grown in DMEM with GlutaMAX, 10% New Born Calf Serum and 1% 100 mM sodium pyruvate in poly-L-ornithine coated T175 flasks without antibotics at 37°C and 5% CO_2_ in air. Cells were incubated with 0 ng/ml (sterile water), 0.1 ng/ml, 1.0 ng/ml, 10.0 ng/ml and 100 ng/ml TNFα (active mouse TNFα full length protein – Abcam (ab9740)) for 30 minutes or 24 hours. Three biological replicates per condition and time point were used. Cells were washed with ice-cold phosphate-buffered saline (PBS). Subsequently, cells were scraped off in ice-cold PBS with phosphatase and protease inhibitors, centrifuged 2 minutes at 300 × g (4°C) and the supernatant was discarded. The cell pellets were dissolved in ice-cold PBS and centrifuged again. The supernatant was discarded and the cell pellets were frozen at −20°C until further processing.

Human neurons were generated by differentiation of neural stem cells (NSCs) that were derived from human embryonic stem cell (XCell Science Inc). Cell expansion and differentiation into mature neurons was performed according to the company guidelines and as previously described [57]. In brief, the differentiation process went through three phases including proliferation, induction and maturation. NSCs were thawed and seeded into Geltrex (1:100) (Life Technologies) coated plates with Neurobasal Medium (Invitrogen) supplemented with 20 ng/ml bFGF (R&D systems), 1× B27 (Invitrogen), 20 mM MEM NEAA (Invitrogen), 20 mM GlutaMax (Invitrogen) and 1% Penicillin/Streptomycin (Invitrogen) and grown for several passages using StemPro^®^ Accutase (Life Technologies) performed under routine cell culture conditions before starting the differentiation process.

For inducing NSCs into neuronal precursors, the proliferating cells were seeded at a density of 20000 cells/cm^2^ into Poly-L-ornithin (PLO) (Sigma Aldrich) and Laminin (Sigma Aldrich) coated dishes in Neurobasal medium supplemented with the factors described above. At day 2, the medium was changed to Neural Induction Basal Medium (XCell Science) with Neuronal Induction Supplement A, B and C (XCell Science) and cells were induced for 6 days with medium change every second day. For the maturation phase, the neuronal precursor cells were grown into PLO/laminin coated dishes in Neuronal Maturation Basal Medium (XCell Science) supplemented with Neuronal Maturation Supplement A at a density of 35.000 cells/cm^2^ and grown for 8 days. During the maturation process, the medium was changed every second day and cells were not passaged allowing them to fully differentiate into mature neurons.

Cells (untransfected, control-transfected or transfected for CREB knockdown) were incubated with 0 ng/ml (sterile water, negative control), 1.0 ng/ml TNFα (active human TNFα full length protein – Abcam (ab83544)) or 50 μM H_2_O_2_ (positive control; 31642, Sigma) for 24 hours. Three biological replicates per condition and time point were used. Cells were washed with ice-cold PBS. Subsequently, cells were scraped off in ice-cold PBS with phosphatase and protease inhibitors, centrifuged 2 minutes at 300 × g (4°C) and the supernatant was discarded. The cell pellet was dissolved in ice-cold PBS and centrifuged again. The supernatant was discarded and the cell pellets were frozen at −20°C until further processing.

### Immunofluorescence

For double immunofluorescence staining, NSCs were grown in 24-well plates containing glass coverslips (VWR) essentially as described above regarding surface coating, medium and cell density. After differentiation, cells were fixed in 4% paraformaldehyde (PFA; Sigma) in PBS for 25 min at room temperature (RT). For gamma-aminobutyric acid (GABA) staining, cells were fixed in 4% PFA and 0.05% glutaraldehyde (Sigma).

Cells were rinsed with 0.05 M Tris-buffered saline (TBS)/0.1% Triton-X-100 (Sigma), then preincubated with TBS/5% goat serum (Gibco) according to the host of the secondary antibody, and incubated with a mixture of two of the following primary antibodies diluted in TBS/5% goat serum for 24 hrs at 4°C. Antibodies were used at the following concentrations: glial fibrillary acidic protein (GFAP; rabbit anti-; DAKO) 1:4000; β-tub III (mouse anti; Sigma) 1:2000; GABA (rabbit anti-; Chemicon) 1:500 and Ki67 (mouse anti-; BD Pharmingen) 1:500 in TBS/5% goat serum. Subsequently, cultures were incubated with a mixture of Alexa Fluor^®^ 555 conjugated anti-mouse IgG and Alexa Fluor^®^ 488 conjugated anti-rabbit IgG at 1:200 for two hrs at RT. Cell nuclei were counterstained with 4′,6-diamidino-2-phenylindole (DAPI; Sigma) at 10 μM in TBS. Cultures were mounted onto glass slides with Prolong^®^ Gold mounting medium (Molecular Probes). Images were recorded using a Zeiss Axiophot epifluorescence microscope connected to a Leica DC300 camera and processed using Adobe^®^ Photoshop^®^ software.

### Transfection of human neurons

RNA-oligonucleotides were used to knockdown CREB protein (Unspecific_AllStars_1 (FlexiTube siRNA premix 1027420, Qiagen) as negative control and Hs_CREB_5 (siRNA5) (SI00299894 – AACCAAGTTGTTGTTCAAGCT, Qiagen) and Hs_CREB_7 (siRNA7) (SI00299908 – AAGCCCAGCCACAGATTGCCA, Qiagen) for knockdown of target protein). Unspecific_AllStars_1 was dissolved in 625 μl RNAse-free water (10 μM stock solution) whereas the siRNAs to knockdown CREB were dissolved in each 100 μl RNAse-free water (10 μM stock solution). 5 μl of each solution was mixed in 250 μl Neuronal maturation media. 15 μl Lipofectamine RNAiMax (13778150, Thermo Fisher Scientific) was dissolved in 250 μl serum-free Neuronal maturation media as well. Subsequently, 250 μl of siRNA-containing media was mixed with 250 μl lipofectamine containing media and incubated for 40 minutes at RT. The final siRNA/FlexiTube siRNA concentration per 10 cm petri dish was 50 pmol (500 μl siRNA-lipid media complex per well) with a total media volume of 10 ml. The transfection of human neurons started 24 hours before stimulation with/without TNFα.

### Cell viability and Caspase 3/7 activity assays

For both HT22 cells and the human stem cell-derived neurons, cell viability and caspase activity were determined using CellTiter-Glo^®^ Cell viability assay and Caspase-Glo^®^ 3/7 assay, respectively (both Promega). Cells were cultured as described above at a density of 2500 or 5000 (HT22) and 7000 cells per well (human neurons) in white polystyrene flat-bottomed 96-well plates. The cells were stimulated with 0.1, 1, 10 or 100 ng/ml TNFα and untreated cells served as control. After 24 hrs of stimulation, CellTiter-Glo or Caspase-Glo reagents were added to each well according to the manufacturer's instructions. After 10 min (CellTiter-Glo) or 30 min (Caspase-Glo) at RT, the luminescence signals were measured against medium (wells without cells) using a BMG fluostar Omega Plate reader (BMG Labtech).

### Reactive oxygen species detection and mitochondrial membrane potential assay

Oxidative stress and mitochondrial membrane potential (MitoMP) were measured for the human stem cell-derived neurons with a reactive oxygen species (ROS) detection assay (Abcam) and a MitoMP assay (Cell Signaling). Cells were cultured as described above at a density of 7000 cells per well in white polystyrene flat-bottomed 96-well plates [58]. The cells were stimulated with 0.1, 1.0, 10.0 or 100.0 ng/ml TNFα and untreated cells served as control. For the ROS assay, after 24 hrs of stimulation the cells were incubated for 60 min at 37°C with the ROS Red Dye working solution. Thereafter, some cells were treated with 10 μM or 20 μM DMSO for 15 min as positive controls whereas the fluorescence signal (Ex490/Em620, bottom optics) was measured against medium (wells without cells) using a BMG fluostar Omega Plate reader (BMG Labtech).

For the MitoMP assay, after 24 hrs of stimulation the cells were incubated with 2 μM JC-1 for 15 min. Some non-stimulated cells were incubated with 50 μM CCCP for 5 min before adding JC-1 serving as positive controls. Thereafter cells were washed with PBS and added PBS before measuring the fluorescence signal (Ex490/Em520) against medium (wells without cells) using a BMG fluostar Omega Plate reader (BMG Labtech).

### Cell lysis and protein reduction

Cell pellets were lysed and reduced in 6 M urea, 2 M thiourea and 10 mM DTT at RT. Afterwards, samples were diluted 10 times by adding 20 mM TEAB, pH 7.5. After vortexing and sonication, a protein aliquot was taken out and stored at −20°C for Western blotting. A total of 60 μg protein per condition were used for mass spectrometry-based proteomics.

### Protein alkylation and enzymatic digestion

A total of 60 μg of proteins were alkylated in 20 mM iodoacetamide (IAA) for 30 minutes in the dark at RT. Subsequently, trypsin (1:50 (w/w) trypsin:protein) was added and the solution was incubated overnight at 37°C. Protein and peptide quantification was performed by fluorometric quantification (Qubit^TM^ – Life Technologies). The peptide solution was dried in a vacuum centrifuge before TMT labelling.

### TMT10-plex labelling

Three and two biological replicates were used for HT22 cells and human neurons, respectively. The labelling was performed according to manufacturer's instruction. The labelled peptides of each condition were mixed 1:1:1:1:1:1:1:1:1:1, dried down and stored for further enrichment and analysis.

### Enrichment and purification of phosphorylated peptides using TiO2

The workflow is described in detail by Larsen et al. [59]. Briefly, the combined labelled peptides (600 μg peptides in total) were dissolved in TiO_2_ loading buffer (80% acetonitrile (ACN), 5% trifluoroacetic acid (TFA) and 1 M glycolic acid) and incubated with 3.6 mg of TiO_2_ (titansphere TiO_2_, 5μm; a kind gift from GL Sciences, Japan) beads for 30 minutes at RT. The beads were sequentially washed with TiO_2_ loading buffer, 80% ACN/1% TFA and 10% ACN/0.1% TFA. Phosphorylated peptides were eluted with 1.5% ammonium hydroxide solution, pH 11.3, and dried. The flow-through was incubated again with TiO_2_ (1.8 mg) and processed as before and the two TiO_2_ beads approaches were combined. The unbound TiO_2_ fraction and the combined washing fractions contain unmodified peptides. All the eluates were dried and desalted on micro-columns before capillary hydrophilic interaction liquid chromatography (HILIC) fractionation.

### Sample desalting with R2/R3 micro-column

The samples were desalted before HILIC fractionation using home-made P200-tip-based columns packed with equal ratios of Poros R2 (Oligo R2 Reversed Phase Resin 1-1112-46, Applied Biosystems) and Poros R3 (OligoR3 Reversed Phase Resin 1-1339-03, Applied Biosystems) reversed-phase resin material. The end of the tip was blocked with C_8_ material (Model 2314, 3 m EmporeTM C8). The column was prepared by short centrifugation (1000× g) of the R3 reversed-phase resin (100% ACN). The column was equilibrated with 0.1% TFA and centrifuged again. The acidified samples were loaded onto the columns and washed / centrifuged three times with 0.1% TFA. Peptides were eluted with 60% ACN, 0.1% TFA and dried.

### HILIC fractionation

The unmodified and phosphorylated peptide samples were fractionated prior to nano liquid chromatography-tandem mass spectrometry (nLC-MS/MS) analysis using HILIC as described previously [60, 61]. Peptides were dissolved in 90% ACN, 0.1% TFA (solvent B) and loaded onto a 450 μm OD × 320 μm ID × 17 cm micro-capillary column packed with TSK Amide-80 (3 μm; Tosoh Bioscience) using an Agilent 1200 Series HPLC (Agilent). The peptides were separated using a gradient from 100–60% solvent B (A = 0.1% TFA) in 30 min at a flow-rate of 6 μl/min. Fractions were collected every 1 min based on the UV chromatogram. Subsequently, the peptide fractions were dried by vacuum centrifugation.

### Reversed-phase nanoLC-ESI-MS/MS

The peptides (resuspended in 0.1% formic acid (FA)) were automatically injected and loaded on a ReproSil-Pur C18 AQ (Dr. Maisch, Ammerbuch-Entringen, Germany) in-house packed trap column (2 cm × 100 μm inner diameter; 5 μm). The peptides were separated at 250 nl/min on an analytical ReproSil-Pur C18 AQ (Dr. Maisch, Ammerbuch-Entringen, Germany) packed in-house column (17 cm × 75 μm; 3 μm) by reversed phase chromatography which was operated on an EASY-nanoLC system (Thermo Fisher Scientific, Odense, Denmark). Mobile phase was 95% ACN/0.1% FA (B) and water/0.1% FA (A). The gradient was from 1% to 30% solvent B in 80 min, 30–50% B in 10 min, 50–100% B in 5 min and 8 min at 100% B. The nano-LC was online connected to a Q Exactive HF Hybrid Quadrupole-Orbitrap mass spectrometer (Thermo Fisher Scientific) operating in positive ion mode and using data-dependent acquisition. The Orbitrap acquired the full MS scan with an automatic gain control (AGC) target value of 3e6 ions and a maximum fill time of 100 ms. Each MS scan was acquired at high-resolution (120,000 full-width half maximum (FWHM) at m/z 200 in the Orbitrap with a mass range of 400–1400 Da. The 12 most abundant peptide ions were selected from the MS for higher energy collision-induced dissociation (HCD) fragmentation (collision energy: 34 V) if they were at least doubly charged. Fragmentation was performed at high resolution (60,000 FWHM) for a target of 1e5 and a maximum injection time of 60 ms using an isolation window of 1.2 m/z and a dynamic exclusion of 20 s.

### Data analysis of mass spectrometry experiments

Raw data were searched against the Swissprot database and Uniprot mouse/human reference database via Mascot (v2.3.02, Matrix Science) and Sequest HT search engines, respectively, using Proteome Discoverer (v1.4.1.14, Thermo Fisher Scientific). A precursor mass tolerance of 10 ppm and a product ion mass tolerance of 0.02 Da were applied allowing not more than one missed cleavage for trypsin. Fixed modifications included carbamidomethylation of Cys and TMT10-plex labeling for Lys and N-terminal. Variable modifications contained phosphorylation on Ser/Thr/Tyr. The TMT10plex datasets were quantified using the centroid peak intensity with the “reporter ions quantifier” node. To ensure a high-confident identification of peptides, we used the Mascot percolator algorithm (*q* value filter set to 0.01), Mascot and Sequest HT peptide rank 1 and a cut-off value of Mascot score ≥ 22 as well as Sequest HT ΔCn of 0.1. Moreover, a cut-off value of Xcorr score for charge states of +1, +2, +3 and +4 higher than 1.5, 2, 2.25 and 2.5, were considered for further analysis. Subsequently, these peptides were filtered against a Decoy database resulting into a false discovery rate (FDR) of 0.01 (FDR < 0.01). Quantification was performed on the log2-values of the measured peptide reporter ion intensities and the data were normalized based on the median. Modified peptides were merged with the R Rollup function (http://www.omics.pnl.gov) allowing for one-hit-wonders and using the mean of the normalized intensities for each peptide. Quantification of proteins was obtained by merging the unmodified peptides with the R Rollup function considering at least 2 unique peptides by not allowing for one-hit-wonders and using the mean of the intensities. Subsequently, the mean over the experimental conditions for each peptide in each replicate was subtracted in order to merge the data from different replicates. PhosphoRS was used to confidently localise phosphorylation sites with a confidence filter of 95%. Phosphorylated peptides were normalized based on the protein expression in each of the replicates including the proteins with only one unique peptide to ensure that deregulation occurred on post-translation modification (PTM) level and not on protein level. Deregulated proteins and phosphorylated proteins were regarded to be significantly deregulated if they fulfilled a *p*-value of <0.01 regarding HT22 cell study (*n* = 3). We applied combined limma and rank product tests [62], subsequently corrected for multiple testing according to Storey. In case of human neurons, a fold-change threshold of ± 1.3 accompanied with a maximal standard deviation of 30% was used (*n* = 2) due to sample generation limitations. These filtering parameters of proteomics data sets were successfully applied recently within *in vitro/in vivo* projects in the field of neuroscience [23, 40, 63].

The mass spectrometry proteomics data have been deposited to the ProteomeXchange Consortium [64] via the PRIDE partner repository with the dataset identifier PXD005675 (username: reviewer88432@ebi.ac.uk, password: nNWGP0Vk).

### Bioinformatics analysis of protein classes and affected signaling pathways

Deregulated proteins were categorised into protein classes using PANTHER (Protein Analysis Through Evolutionary Relationships) classification system software (http://www.pantherdb.org) and the general annotation from UniProt (http://uniprot.org). The analyses of affected signaling pathways from all deregulated proteins and PTM profile of proteins were performed independently with the INGENUITY Pathway Analysis [43] (http://www.ingenuity.com) software tool that comprises curated information from databases of experimental and predictive origin, enabling discovery of highly represented functions and pathways. The mean of the ratios of all deregulated phosphorylated peptides per protein was used for signaling pathway analysis. Network analysis was performed by uploading separately the deregulated unmodified and phosphorylated proteins. We used only the database information of experimental and predictive origin regarding central nervous system to be confident about the potential affected signaling pathways. The IPA comparison analysis takes into account the signaling pathway rank according to the calculated *p*-value and reports it hierarchically. The software generates significance values (*p*-values) between each biological or molecular event and the imported proteins based on the Fisher's exact test (*p* ≤ 0.05). Upstream regulator analysis performed by IPA software was used to get more information about pathway nodes that are overlapping by the uploaded deregulated lists of found hits. A z-score greater 2 or smaller than −2 was used as significance level.

### Quantification of proteins and phosphorylation-motifs by immunoblotting

Protein extracts (15 μg) were separated on 4–12% Bolt Bis-Tris gradient gels (NW04125Box, Novex) and transferred to polyvinylidene fluoride (PVDF) membranes via the TransBlot SD Semi-Dry Transfer Cell Blotter system (Biorad). The protein content on the membranes was visualised with Ponceau S. The membranes were incubated in blocking buffer (5% milk) washed and incubated overnight at 4°C with primary antibody dilutions as recommended by the manufacturer (Phospho-PKC Substrate Motif (R/KXpSX(R/K) MultiMab Rabbit Monoclonal Antibody – #6967, Cell Signaling; Phospho-PKA Substrate (RRXS*/T*) (100G7E) Rabbit Monoclonal Antibody - #9624, Cell Signaling; rabbit polyclonal antibody against p44/42 MAPK (Erk1/2) - #9102, Cell Signaling; rabbit polyclonal antibody against phospho-p44/p42 MAPK (Erk1/2) (Thr202/Tyr204) - #9101, Cell Signaling; goat polyclonal antibody against GAPDH – ab9483, Abcam; murine monoclonal antibody against CREB (clone 86B10) - #9104, Cell Signaling; rabbit monoclonal antibody against phospho-CREB (Ser133) (clone 87G3) - #9198, Cell Signaling; total OXPHOS murine monoclonal antibody cocktail against CV-ATP5A, CIII-UQRC2, CII-SDHB and CI-NDUFB8 – ab110413, Abcam).

The blots were washed and incubated with horseradish peroxidase-conjugated secondary antibody (anti-rabbit IgG, HRP-linked antibody - #7074, Cell Signaling, anti-mouse IgG, HRP-linked antibody – ab6728, Abcam or anti-goat IgG, HRP-linked antibody – ab6741, Abcam) in 5% milk or 3% bovine serum albumin (BSA; for phospho antibodies) for 2 hours. Subsequently, blots were washed and developed with ECL system (Luminata^TM^ Forte Western HRP Substrate, WBLUF0100, Millipore) using standard protocol from the manufacturer. Immunoblots were considered for quantification if (i) the pattern and intensity of lanes stained with PonceauS were equal and total lane intensity of the PonceauS-stained proteins was similar within the biological replicates. For phospho-motif immunoblots, the total lane intensity was selected as a representative indicator of global changes in PKA- and PKC-phospho motifs. Immunoblots were quantified with TotalLab TL100 software (www.totallab.com) using software-suggested background correction. Each band was normalised against the total lane intensity obtained by Ponceau S or against GAPDH that was not changed during our proteomics analysis. Fold-changes were calculated between TNFα-stimulated and control samples. Three biological replicates were used for statistical analysis (Student's *t*-test, unpaired) with a significance threshold of 0.05.

### Isolation of total RNA and pathway-focused gene expression analysis related to synaptic plasticity via quantitative PCR

Total RNA from human neurons (control and 1.0 ng/ml TNFα for 24 hours; *n* = 3) was isolated and purified by mirVana^TM^ Isolation Kit (Ambion) according to the manufacturer's instructions. Total RNA was eluted with nuclease-free water. The optical density (OD) ratio of 260/280 was measured using a Nanodrop spectrophotometer (PeqLab Biotechnology); it ranged between 1.9 and 2.1. Eluates were stored at −20°C until further analysis.

The RNA isolates (100 ng) were used to quantify the gene expression of 84 mRNA transcripts related to synaptic plasticity (RT2 Profiler Mouse Synaptic Plasticity – PAMM-126Z, Qiagen) according to the manufacturer's protocol on a StepOnePlus device (Applied Biosystems). The relative expression of each mRNA was normalised against the average Ct value of 5 reference genes (ACTB, B2M, GAPDH, HPRT1 and RPLP0) using the equation 2^−ΔΔCt^, where ΔΔCt = ΔCt_TNFα_ – ΔCt_control_ and ΔCt = Ct_target-mRNA_ – Ct_average-of-5-reference-genes_. Gene expression changes were considered significant if they reached a *p*-value of ≤ 0.05 (Student's *t*-test, unpaired). Three biological replicates were used in each group (control and 1.0 ng/ml TNFα).

### Behavioural and molecular analysis of TNFα knockout and C57BL/6 mice

#### Mice

For this experiment all mice used were 9-month-old female mice. TNFα knockout mice [8, 65] were purchased at Jackson Laboratories (USA) and bred on a C57BL/6 background. Female C57BL/6 mice were purchased from Taconic A/S (Denmark) except from a group of 9-month-old C57BL/6 mice, which was purchased from Harlan Laboratories (Netherlands). Mice were housed in the Biomedical Laboratory at the University of Southern Denmark. All experiments were conducted under permission from the Danish Ethical Animal Care Committee (Permissions no. 2011/562-67 and 2011/561-1950).

#### Barnes maze experiment - LPS treatment

The effect of TNFα on the learning ability was studied in TNFα knockout (*n* = 11) mice using C57BL/6 mice (*n* = 16) as controls. Neuroinflammation was induced by intraperitoneal injection of TNFα knockout (*n* = 8) and C57BL/6 mice (*n* = 8) with lipopolysaccharide (LPS) (Serum E-coli 0111:B4, Sigma) injections (i.p) at a dose of 0.5 mg/kg at day 7 and 4 prior to learning.

#### Barnes maze experiment - behaviour analysis

The Barnes maze [66] was used to study the ability of C57BL/6 and TNFα knockout mice to learn during the acquisition phase as described recently [67]. The Barnes maze is a 92 cm diameter maze with 20 holes distributed around the perimeter (Panlab Spain) and the testing was performed under a high power lamp source to motivate the mice. Two weeks prior evaluation of mice on the Barnes maze, the mice were individually housed and placed in a room reserved to behavioural assessment. Mice were tested in the Barnes maze for 6 consecutive days with 4 trials per day. The location of the goal box and distal spatial cues were constant during the experiment. Each mouse was placed within a cylinder in the middle of the maze for 30 seconds. The mouse was then allowed 4 minutes to find the goal box and, if not found within 4 minutes, the mouse was gently guided towards the goal box by hand. When the mouse located the goal box itself, it was kept in timeout in the box for the remaining time of the 4 minutes. The latency time is the time the mouse spends on the maze before finding the goal box. The maze was cleaned with 70% ethanol between each mouse. Statistical analysis was performed using Prism (GraphPad Software, version 6). Data are presented as mean ± standard error of the mean. Data were analysed using repeated measures (RM) two-way analysis of variance (ANOVA) followed by Sidak's Multiple Comparison Test. Differences were considered significant if *P* ≤ 0.05. *P*-values are indicated as follows: *P* ≤ 0.05: *, *P* < 0.01: **, *P* < 0.001: ***, and *P* < 0.0001: ****.

#### Histology

Mice were euthanized by an overdose Pentobarbital following by transcardial perfusion with 5 ml PBS and 20 ml 4% PFA. The brains were thereafter placed in 20% sucrose overnight, and frozen. Brains were stored at −20°C until they were sectioned in a cryostate into 35 mm thick coronal. Parallel sections spanning the entire forebrain were stained with toluidine blue [8].

#### CREB and p-CREB immunoblotting of hippocampus

Three-month-old C57BL6 wild-type and TNFα knockout littermate mice were used to explore the connection between TNFα and CREB signaling under physiological and inflammatory conditions (*n* = 3–6/treatment group & genotype, *n* = 17). Mice received a single intraperitoneal injection with either sterile PBS or 0.5 mg/kg LPS (Serum E-coli 0111:B4, Sigma, Denmark) in sterile PBS (injection volume ∼150 μL). Four hours after the injections, the mice were killed by cervical dislocation and their brains were immediately removed and dissected on a Petri dish filled with ice. The left hippocampus was heat-stabilized using the StabilizorTM T1 (Denator), and subsequently frozen on dry-ice for immunoblotting as described earlier.

## CONCLUSIONS

This study shows that several molecular targets and signaling pathways induced by TNFα in neurons resemble those of AD pathology. It may suggest that TNFα functions as a contributing factor to this neurodegenerative disease but at the same time has neuroprotective properties regulating learning and memory formation under neuroinflammatory conditions resembling those noted in AD.

## SUPPLEMENTARY FIGURES AND TABLES





## Abbreviations

AMPA: α-amino-3-hydroxy-5-methyl-4-isoxazolepropionic acid
AD: Alzheimer disease
ANOVA: analysis of variance
AGC: automatic gain control
ACN: acetonitrile
BSA: bovine serum albumin
FA: formic acid
FDR: false discovery rate
FWHM: full-width half maximum
GABA: gamma-aminobutyric acid
HILIC: hydrophilic interaction liquid chromatography
HCD: higher energy collision-induced dissociation
IPA: Ingenuity Pathway Analysis
IAA: iodoacetamide
LPS: lipopolysaccharide
LC-MS/MS: liquid chromatography-tandem mass spectrometry
MitoMP: mitochondrial membrane potential
NMDA: N-methyl-D-aspartate
NSCs: neural stem cells
OD: optical density
PBS: phosphate buffered saline
PLO: poly-L-ornithin
PVDF: polyvinylidene fluoride
PFA: paraformaldehyde
PTM: post-translation modification
RM: repeated measures
RT: room temperature
ROS: reactive oxygen species
SEM: standard error of the mean
TFA: trifluoroacetic acid
TBS: Tris-buffered saline
