# Supplementary material for: TNFα affects CREB-mediated neuroprotective signaling pathways of synaptic plasticity in neurons as revealed by proteomics and phospho-proteomics
Source: Oncotarget. 2017 Jul 21;8(36):60223–42. doi: 10.18632/oncotarget.19428 (PMC5601134; doi:10.18632/oncotarget.19428)
Supplement: Supplementary file 1 [file oncotarget-08-60223-s001.pdf]

TNFα affects CREB-mediated neuroprotective signaling pathways of synaptic plasticity in neurons as revealed by proteomics and phospho-proteomics

SUPPLEMENTARY MATERIALS

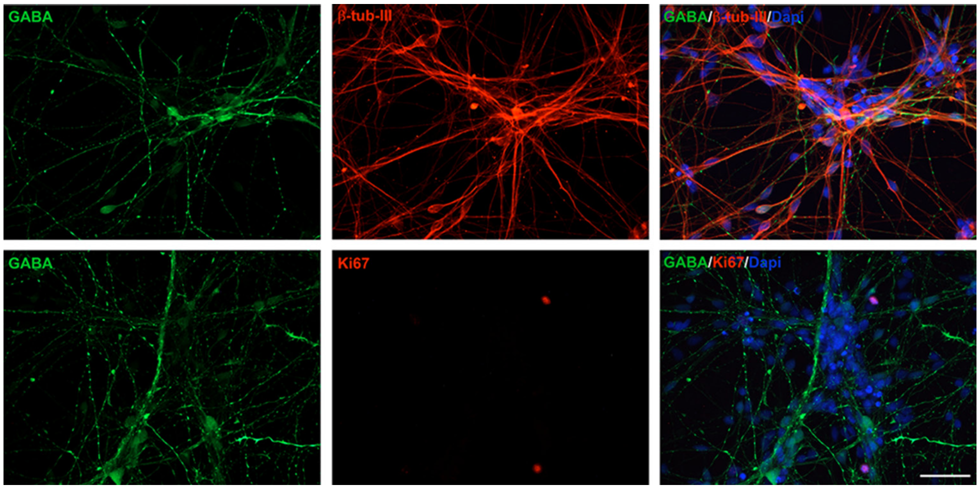

Supplementary Figure 1: Differentiated human neuronal stem cells. The panel shows that the differentiation protocol of human neuronal stem cells into mature neurons resulted in a population with GABAergic neurons (GABA - green) and only few proliferating cells (Ki67 - red) (lower panels). Cytoskeleton stained with β-tubulin III (ß-tub-III - red) is shown in the upper panels. Nuclear chromatin was stained with DAPI; bar = 50 μM.

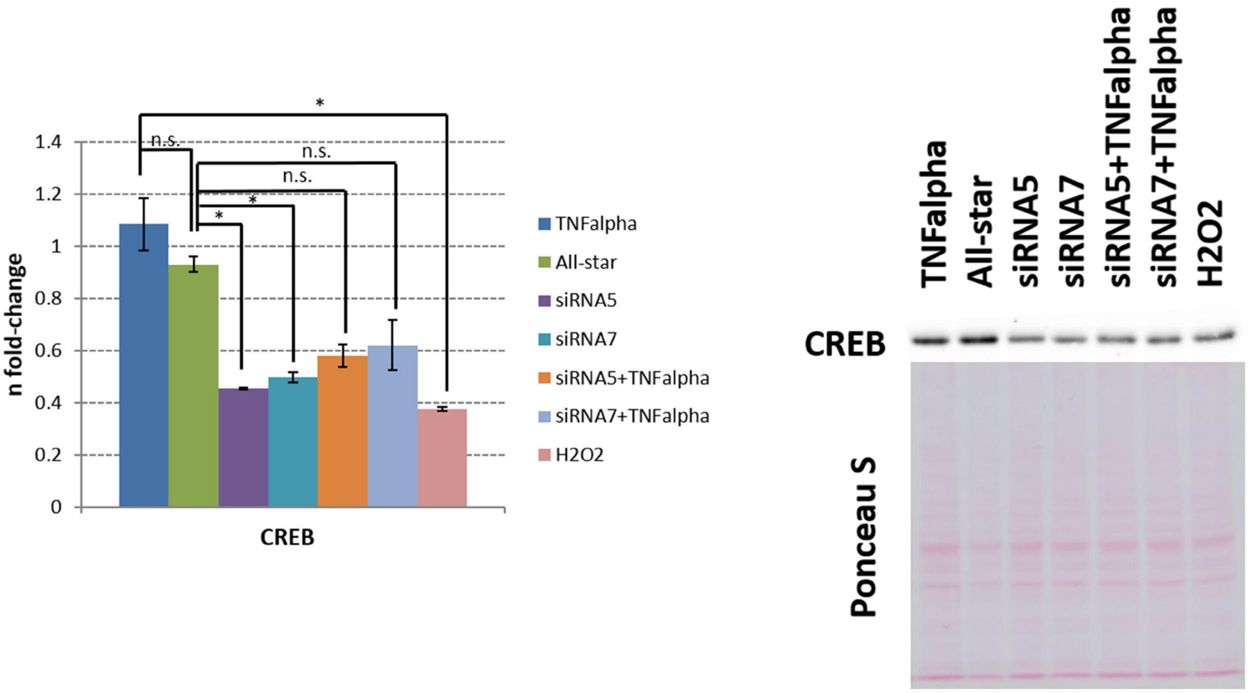

Supplementary Figure 2: Immunoblotting of CREB protein expression in human neurons. Data from CREB immunoblot (human neurons, TNFα stimulation over 24 hours) is shown with following groups: TNFα-dose of 1 ng/ml alone, Allstar as transfection control, siRNA5 and siRNA7 against CREB expression, siRNA5 and siRNA7 knockdown prior to 24 hours TNFα (1.0 ng/ml) stimulation and H_2O_2 incubation serving as positive control. The columns represent the fold-changes with standard errors of the mean [67]; $n = 2$; $*p < 0.05$; $**p < 0.01$; $***p < 0.001$, n.s; not significant (unpaired Student s *t*-test). Normalization was performed against total lane intensity via Ponceau S staining.

Supplementary Table 1: Identifiable list of unmodified proteins from proteomics experiments with HT22 cells and TNFα stimulation. See Supplementary_Table_1

Supplementary Table 2: Identifiable list of phospho-proteins with peptide sequence from proteomics experiments with HT22 cells and TNFα stimulation. See Supplementary_Table_2

Supplementary Table 3: Quantifiable list of unmodified proteins from proteomics experiments with HT22 cells and TNFα stimulation. See Supplementary_Table_3

Supplementary Table 4: Quantifiable list of phospho-proteins with peptide sequence from proteomics experiments with HT22 cells and TNFα stimulation. See Supplementary_Table_4

Supplementary Table 5: Significantly deregulated unmodified proteins and phospho-proteins from proteomic experiments of HT22 cells with TNFα stimulation. See Supplementary_Table_5

Supplementary Table 6: Identifiable list of unmodified proteins from proteomics experiments with human neurons and TNFα stimulation and/or siRNA knockdown of CREB. See Supplementary_Table_6

Supplementary Table 7: Identifiable list of phospho-proteins from proteomics experiments with human neurons and TNFα stimulation and/or siRNA knockdown of CREB. See Supplementary_Table_7

Supplementary Table 8: Quantifiable list of unmodified proteins from proteomics experiments with human neurons and TNFα stimulation and/or siRNA knockdown of CREB. See Supplementary_Table_8

Supplementary Table 9: Quantifiable list of phospho-proteins from proteomics experiments with human neurons and TNFα stimulation and/or siRNA knockdown of CREB. See Supplementary_Table_9

Supplementary Table 10: Significantly deregulated unmodified proteins and phospho-proteins from proteomic experiments with human neurons and TNFα stimulation and/or siRNA knockdown of CREB. See Supplementary_Table_10

Supplementary Table 11: Proteomic data of CREB protein expression in human neurons experiments. See Supplementary_Table_11

Supplementary Table 12: Analysis of the pathway-focused transcriptome associated to synaptic plasticity in human neurons stimulated with 1.0 ng/ml TNFα over 24 hours and controls. See Supplementary_Table_12
